# Expert consensus to optimize the treatment of elderly patients with luminal metastatic breast cancer

**DOI:** 10.1007/s12094-021-02766-8

**Published:** 2022-02-01

**Authors:** M. D. Torregrosa-Maicas, S. del Barco-Berrón, A. Cotes-Sanchís, L. Lema-Roso, S. Servitja-Tormo, R. Gironés-Sarrió

**Affiliations:** 1 Oncogeriatric Section, Sociedad Española de Oncología Médica (SEOM), Doctor Peset University Hospital, Avda. de Gaspar Aguilar, 90, 46017 Valencia, Spain; 2 Oncogeriatric Section, Sociedad Española de Oncología Médica (SEOM), Doctor Josep Trueta University Hospital, Insituto Catalán de Oncología (ICO), Girona, Spain; 3 Oncogeriatric Section, Sociedad Española de Oncología Médica (SEOM), Elda-Virgen de La Salud University Hospital, Alicante, Spain; 4 Grupo Cooperativo de Investigación Clínica en Cáncer de Mama (SOLTI), Doce de Octubre University Hospital, Madrid, Spain; 5 Grupo Español de Investigación en Cáncer de Mama (GEICAM), Hospital del Mar-Parc de Salut Mar, Barcelona, Spain; 6 Oncogeriatric Section, Sociedad Española de Oncología Médica (SEOM), La Fe University Hospital, Valencia, Spain

**Keywords:** Cyclin inhibitors, Geriatrics, Hormone therapy, mTOR inhibitors, PARP inhibitors, PI3K inhibitors

## Abstract

Most patients diagnosed with luminal metastatic breast cancer (MBC) who are seen in oncology consultations are elderly. MBC in elderly patients is characterized by a higher percentage of hormone receptor (HR) expression and a lower expression of human epidermal growth factor receptor 2 (HER2). The decision regarding which treatment to administer to these patients is complex due to the lack of solid evidence to support the decision-making process. The objective of this paper is to review the scientific evidence on the treatment of elderly patients with luminal MBC. For this purpose, the Oncogeriatrics Section of the Spanish Society of Medical Oncology (SEOM), the Spanish Breast Cancer Research Group (GEICAM) and the SOLTI Group appointed a group of experts who have worked together to establish consensus recommendations to optimize the treatment of this population. It was concluded that the chronological age of the patient alone should not guide therapeutic decisions and that a Comprehensive Geriatric Assessment (CGA) should be performed whenever possible before establishing treatment. Treatment selection for the elderly population should consider the patient’s baseline status, the expected benefit and toxicity of each treatment, and the impact of treatment toxicity on the patient’s quality of life and functionality.

## Introduction

In the coming years, an increase in the elderly population, especially those at the most advanced ages, is expected [1]. Cancer is a disease associated with ageing; therefore, it is expected that a considerable increase in the number of cancer cases in elderly patients will occur. Breast cancer (BC) is the most common tumour in women, and its incidence increases with age. Approximately half of all BC cases occur in women older than 65 years [2].

Nowadays, there is not a consensus definition of elderly patient. In the context of clinical trials, patients older than 65 years old are usually considered elderly; however, in the clinical practice, these patients are at least over 70 years of age. Apart from age, a geriatric evaluation would be needed to correctly define an elderly patient. Breast tumours in older patients have certain characteristics that differentiate them from those of younger patients [3, 4]. From a biological perspective, these tumours have a higher percentage of hormone receptor (HR) expression and lower expression of human epidermal growth factor receptor 2 (HER2). From a clinical perspective, older patients are diagnosed with more advanced tumours [4]. On many occasions, elderly patients do not receive adequate curative treatment, which increases the risk of recurrence. Together, these circumstances mean that there will be a high percentage of elderly patients with advanced disease who will need to be offered the best possible treatment [4].

The decision regarding which treatment to administer to older patients with cancer is complex. Concerns about toxicity increase the risk that treatments with proven efficacy will be rejected, with a consequent effect on patient survival and quality of life [5]. An added difficulty is the scarcity of scientific evidence regarding the efficacy and toxicity of cancer treatments in the elderly population, which means that solid evidence to support decision-making is limited [6].

The objective of this paper was to review the scientific evidence on the treatment of elderly patients with luminal metastatic breast cancer (MBC). For this purpose, the Oncogeriatrics Section of the Spanish Society of Medical Oncology (Sociedad Española de Oncología Médica [SEOM]), the Spanish Breast Cancer Research Group (Grupo Español de Investigación en Cáncer de Mama [GEICAM]) and the SOLTI Group appointed a group of experts who have worked together to establish consensus recommendations that will allow the optimization of treatment for elderly patients with luminal MBC.

## Assessment of the geriatric population with breast cancer

People age differently; therefore, chronological age does not always coincide with biological age [7]. Elderly individuals may have health problems that are undetectable in a traditional medical interview, but which may limit the success of a treatment, or cause side effects that increase an individual’s functional or cognitive decline. The American Society of Clinical Oncology (ASCO) [8, 9], the European Society of Medical Oncology (ESMO) [10], SEOM, and the International Society of Geriatric Oncology (SIOG) all support the use of scales that assess the health status, cognitive status and life expectancy of patients with cancer before deciding on a medical intervention [11, 12]. However, SEOM recommends performing a comprehensive geriatric assessment (CGA) only when resources are available, considering time availability in the office and the oncologist’s training in geriatrics. In this regard, the SIOG has published several guidelines for the management of BC in elderly women and for the treatment of side effects caused by treatment [13, 14].

CGA is a multidimensional and interdisciplinary diagnostic process that quantifies and describes undetected medical problems and the functional, mental, social and emotional status of patients. In addition, it aids in the distribution of resources, determining the need for services and developing a plan for preventive, therapeutic, rehabilitative and long-term follow-up care [12]. The information obtained allows the biological age of patients to be determined and treatment to be personalized to improve patients’ functional status and health outcomes. Therefore, the authors of this consensus consider that a CGA adds complete and objective information on the patient at all dimensions, so that the decision does not rely exclusively on the oncologist’s perception on how the treatment will be tolerated.

The geriatric assessment process consists of the following three steps: (i) selection of patients who may benefit from a CGA after a previous screening; (ii) the CGA and treatment plan; and iii) implementation and adherence to recommendations. Several domains are included in the CGA (Table 1) [15]. The assessment of functional status evaluates how the disease or deficit affects the patient and his or her environment and social context. A cognitive and affective, sociofamilial and nutritional assessment is also performed. Patients’ pharmacological history, geriatric syndromes and comorbidities are described. In addition, some components of the CGA can help to predict the survival of elderly patients with cancer and determine the toxicity that could result from treatment.

**Table 1:** CGA with recommended scales [84]

Functional status	Barthel Index Lawton-Brody Scale Gait speed test
Cognitive function	Pfeiffer test MMSE
Nutritional status	Mini Nutritional Assessment
Psychological evaluation and mood	Yesavage scale
Socio-family status	Gijon scale
Comorbidity	Charlson Index
Geriatric syndromes	Fall within the last 6 months Constipation Urinary or faecal incontinence Loss of visual or hearing acuity Insomnia Pressure ulcers Number of prescribed medications
Life expectancy estimate	Onco-MPI Walter Index Suemoto Index
Chemotherapy toxicity prediction	CARG score CRASH CARG-BC score

*CARG* Cancer and Aging Research Group, *CARG-BC* CARG-Breast Cancer, *CGA* comprehensive geriatric assessment, *CRASH* Chemotherapy Risk Assessment Scale for High-age patients, *MMSE* Mini-Mental State Exam, *Onco-MPI* Onco-Multidimensional Prognostic Index

Different tools are available to calculate life expectancy: (i) the oncological‑multidimensional prognostic index (Onco-MPI) [16], which classifies patients with cancer into three groups and estimates the 1-year mortality of each group (0.00–0.46 points: low risk; 0.47–0.63: intermediate risk; and 0.64–1.00: mortality risk > 80%. In the latter case, the use of chemotherapy would be rejected); (ii) the Walter index (https://eprognosis.ucsf.edu) for hospitalized patients ≥ 70 years [17]; and (iii) the Suemoto index for outpatients ≥ 60 years, which estimates the 10-year mortality [18]. The latter two indices can be used to predict mortality risk regardless of whether the patient has cancer. There are also histograms of life expectancy for each age group for Americans without cancer [19].

Regarding the prediction of toxicity, the Cancer and Ageing Research Group (CARG) scale and the Chemotherapy Risk Assessment Scale for High-Age Patients (CRASH) are available [20, 21]. Both differ in the type of toxicity they predict, the populations included in their validation and the predictor variables used. In general, the CARG scale is the most widely accepted. However, it does not predict the toxicity of immunotherapy or hormonal or biological therapies. The CARG-Breast Cancer (CARG-BC) scale was recently developed to predict the toxicity of adjuvant chemotherapy in patients with BC [22].

Using the above instruments, patients can be stratified into risk categories to individualize therapeutic decisions. Frailty is a vulnerability that causes a reduction of the homeostatic reserve and increases the risk of adverse events. Classifications based on frailty have been developed to aid in the decision-making, including those of Balducci, SIOG and Ferrat [23–25]. The classification method that best discriminates 1-year mortality is that of SIOG [24], but all have good prognostic performance for both outpatients and hospitalized patients. The SIOG classification method stratifies patients into three groups according to which treatments can be individualized (Table 2) [24].

**Table 2:** Classification of the health status of geriatric patients for decision making [24]

Healthy	Vulnerable	Frail
Standard treatment	Standard treatment with geriatric intervention	Adapted treatment
G8 ≥ 14	G8 < 14	G8 < 14
CIRS-G grade 0, 1 and 2	CIRS-G at least one grade 3	CIRS-G at least one grade 4
Independent in ADL	Lawton-Brody Scale > 7	Lawton-Brody Scale ≤ 7
	MMSE ≥ 27	MMSE < 24
	Barthel Index 4–5	Barthel Index ≤ 3
No malnutrition	Malnutrition risk	Severe malnutrition

*G8* Geriatric 8 screening tool score, *ADL* activities of daily living, *CIRS-G* Cumulative Illness Rating Scale-Geriatric, *MMSE* Mini-Mental State Examination

## Treatment of elderly patients with luminal metastatic breast cancer

Treatment for elderly patients with luminal MBC aims to improve quality of life and, if possible, increase survival. National and international clinical guidelines recommend prioritizing the use of hormonal treatment because it has a better toxicity profile [26, 27].

Currently, there are several therapeutic strategies for treating HR-positive MBC. Endocrine treatment can be used as monotherapy, or in combination with therapies directed at targets acting on the resistance pathways to hormonal treatment; the targeted therapies include everolimus, a mammalian target of rapamycin (mTOR) inhibitor; phosphoinositol 3-kinase pathway (PI3K) inhibitors, such as alpelisib; and in particular cyclin-dependent kinases 4/6 (CDK4/6) inhibitors such as palbociclib, ribociclib and abemaciclib. These strategies have been shown to increase disease-free survival (DFS) and overall survival (OS) as well as to improve quality of life. However, like all antineoplastic treatments, they are not exempt from toxicity, and their economic cost is very high.

Although there are no specific clinical trials that evaluate these new combinations in the elderly population, data on older patient subgroups in clinical trials, as well as data extracted from real-life studies, can shed light on their efficacy and safety.

### Evidence regarding CDK4/6 inhibitors

Cyclin-dependent kinases regulate cell cycle progression, and CDK4/6 induces the hyperphosphorylation of the retinoblastoma protein, which causes the progression of tumour cells from the G1 checkpoint to the S phase of the cell cycle [28]. The development of resistance to endocrine treatment in BC is associated with dysregulation of the cyclin D/CDK4/6/retinoblastoma pathway [29].

CDK4/6 inhibitors (palbociclib, ribociclib and abemaciclib) are orally administered drugs that, when combined with endocrine therapy as a first-line treatment for patients with HR-positive, HER2-negative MBC, have been shown to increase both progression-free survival (PFS) and OS compared to an aromatase inhibitor (AI) [30–34] or fulvestrant in monotherapy [35–40].

It is important to mention that in these studies, the population ≥ 75 years had limited representation, and all participants had an Eastern Cooperative Oncology Group (ECOG) functional status of 0–1 [41]. The CDK4/6 inhibitors differed in terms of toxicity, a key factor when treating the elderly population. The incidence of neutropenia with the administration of palbociclib and ribociclib stands out, while diarrhoea was the most frequent adverse event associated with the use of abemaciclib. In addition, it is essential to know the usual medications that patients are taking before administering these drugs to prevent pharmacological interactions. The data available to date indicate that advanced age is not a criterion for modifying the dosage of any CDK4/6 inhibitor.

Table 3 summarizes the main efficacy results of the different CDK4/6 inhibitors when used in combination with an AI or fulvestrant, and Table 4 shows the main adverse events observed during treatment with CDK4/6 inhibitors.

**Table 3:** Efficacy of CDK4/6 inhibitors in combination with AI or fulvestrant

Palbociclib [42]	< 65 years		65–74 years		≥ 75 years	
± letrozole (PALOMA-1 and 2)	L	L + P	L	L + P	L	L + P
*N*	183	310	94	162	26	56
PFS, months	12.3	22.0	21.8	27.5	10.9	NR
HR (CI 95%)	0.50 (0.40–0.64)		0.66 (0.45–0.97)		0.31 (0.16–0.61)	
*p*	< 0.001		< 0.016		< 0.001	

± fulvestrant (PALOMA-3)	F	F + P	F	F + P	F	F + P
*N*	131	261	37	59	6	27
PFS, months	5.4	10.9	3.7	16.1	7.4	13.6
HR (CI 95%)	0.59 (0.46–0.75)		0.27 (0.16–0.48)		0.59 (0.19–1.80)	
*p*	< 0.001		< 0.001		< 0.18	

Ribociclib [32]	< 65 years		≥ 65 years	
± letrozole (MONALEESA-2)	L	L + R	L	L + R
*N*	189	184	145	150
PFS, months	13.0	NR	18.4	NR
HR (CI 95%)	0.52 (0.38–0.72)		0.61 (0.39–0.94)	
Interaction *p* value	0.589			

Abemaciclib [46]	< 65 years		65–74 years		≥ 75 years	
± fulvestrant (MONARCH-2)	F	F + A	F	F + A	F	F + A
*N*	133	291	60	114	30	41
PFS, months	10.8	17.4	8.1	14.4	5.8	13.9
HR (CI 95%)	0.52 (0.40–0.68)		0.63 (0.43–0.94)		0.62 (0.34–1.11)	
Interaction *p* value	0.695					

± letrozole o anastrozole (MONARCH-3)	AI	AI + A	AI	AI + A	AI	AI + A
*N*	91	180	54	106	20	42
PFS, months	14.0	27.5	24.2	28.2	9.1	31.1
HR (CI 95%)	0.48 (0.35–0.67)		0.64 (0.40–1.02)		0.54 (0.26–1.13)	
Interaction *p* value	0.634					

*A* abemaciclib, *AI* aromatase inhibitors, *CDK4/6* cyclin-dependent kinase 4 and 6, *CDKi* cyclin-dependent kinase inhibitor, *CI* confidence interval, *F* fulvestrant, *HR* hazard ratio, *L* letrozole, *NR* not reached, *P* palbociclib, *PFS* progression-free survival, *R* ribociclib

**Table 4:** Safety of CDK4/6 inhibitors in combination with AI or fulvestrant

Palbociclib [42]	< 65 years (*N* = 568)		65–74 years (*N* = 221)		≥ 75 years (*N* = 83)	
+ letrozole o fulvestrant PALOMA-1, 2 and 3	G1–4 *n* (%)	G3–4 *n* (%)	G1–4 *n* (%)	G3–4 *n* (%)	G1–4 *n* (%)	G3–4 *n* (%)
Neutropenia	459 (81)	373 (66)	170 (77)	140 (63)	75 (90)	61 (74)
Anaemia	140 (25)	24 (4)	66 (30)	10 (5)	36 (43)	7 (8)
Fatigue	225 (40)	9 (2)	91 (41)	7 (3)	31 (37)	6 (7)
Thrombocytopenia	100 (18)	11 (2)	47 (21)	4 (2)	21 (25)	2 (2)
Infection	296 (52)	22 (4)	138 (62)	20 (9)	50 (60)	6 (7)

Ribociclib [32]	< 65 years (*N* = 184)		≥ 65 years (*N* = 150)	
+ letrozole MONALEESA-2	G1–4 *n* (%)	G3–4 *n* (%)	G1–4 *n* (%)	G3–4 *n* (%)
Neutropenia	137 (75)	108 (59)	111 (74)	90 (60)
Nausea	92 (50)	4 (2)	80 (53)	4 (3)
Diarrhoea	56 (30)	1 (1)	61 (41)	3 (2)
Fatigue	67 (36)	5 (3)	55 (37)	3 (2)
Elevated liver enzymes	34 (19)	18 (10)	26 (17)	14 (9)

Abemaciclib [46]	< 65 years (*N* = 466)		65–74 years (*N* = 219)		≥ 75 years (*N* = 83)	
+ letrozole, anastrozole or fulvestrant MONARCH-2 y 3	G1–4 *n* (%)	G3–4 *n* (%)	G1–4 *n* (%)	G3–4 *n* (%)	G1–4 *n* (%)	G3–4 *n* (%)
Diarrhoea	396 (85)	46 (10)	183 (84)	28 (13)	71 (86)	16 (19)
Neutropenia	215 (46)	120 (26)	106 (48)	60 (27)	25 (30)	15 (18)
Thromboembolic events	19 (4)	9 (2)	11 (5)	6 (3)	11 (13)	4 (5)
Pneumonitis	16 (3)	4 (1)	7 (3)	3 (1)	3 (4)	0 (0)
Liver toxicity^a^	76 (16)	23 (5)	33 (15)	12 (6)	7 (8)	4 (5)

*AI* aromatase inhibitors, *ALT* alanine aminotransferase, *CDK4/6* cyclin-dependent kinase 4 and 6

^a^ALT increase

#### Palbociclib

Palbociclib is the first selective CDK4/6 inhibitor approved by the European Medicines Agency (EMA). Its approval was based on the results of two phase III trials: PALOMA-2 evaluated the use of letrozole with or without palbociclib in patients with MBC who had not received previous systemic treatment [31], and PALOMA-3 compared the use of fulvestrant with or without palbociclib in the pre- and postmenopausal population, without limiting the number of previous lines of endocrine therapy used [35]. Both studies achieved their main objective since a significant increase in PFS was obtained with the addition of palbociclib.

In a subsequent meta-analysis of the PALOMA trials that also included the phase I/II PALOMA-1 trial (letrozole with or without palbociclib as the first-line treatment) [30], it was observed that of the 528 patients treated with palbociclib and letrozole, 41% were ≥ 65 years old, while of the 347 patients treated with palbociclib and fulvestrant, 25% were ≥ 65 years old [42]. In the subgroup of patients ≥ 65 years, a statistically significant increase in PFS was observed for the palbociclib arms. In PALOMA-1/2, among patients aged of 65–74 years, PFS was 27.5 vs. 21.8 months [hazard ratio (HR) 0.66; 95% CI 0.45–0.97; *p* = 0.016] and in patients aged ≥ 75 years, PFS was not reached vs. 10.9 months (HR 0.31; 95% CI 0.16–0.61; *p* < 0.001). In PALOMA-3, among patients aged 65–74 years, the PFS was 16.1 vs. 3.7 months (HR 0.27; 95% CI 0.16–0.48; *p* < 0.001), while in patients ≥ 75 years, it was 13.6 vs. 7.4 months (HR 0.59; 95% CI 0.19–1.8; *p* < 0.18) (Table 3). Recently, the OS data of the PALOMA-3 trial were reported at the 2021 ASCO Annual Meeting after a follow-up of more than 6 years; they indicated that the combination of palbociclib and fulvestrant offered benefits superior to those of monotherapy (34.8 vs. 28.0 months), without specifying the results for the elderly population [40].

Regarding adverse events of any grade, elderly patients had a higher incidence of anaemia (≥ 75 years: 43%; 65–74 years: 30% and < 65 years: 25%), thrombocytopenia (≥ 75 years: 25%; 65–74 years: 21% and < 65 years: 18%), leukopenia (≥ 75 years: 55%; 65–74 years: 43% and < 65 years: 48%) and neutropenia (≥ 75 years: 90%; 65–74 years: 77% and < 65 years: 81%). Neutropenia occurred most frequently with the use of palbociclib. However, the incidence of febrile neutropenia was very low and was similar for all age groups (≥ 75 years: 2%; 65–74 years: 1% and < 65 years: 1%) [37]. It is important to note that neutropenia secondary to CDK4/6 inhibitor use is the result of the arrest of the cell cycle and not the death of neutrophil proliferation precursors, as occurs with chemotherapeutic agents. This adverse effect is controlled with the temporary suspension of the drug and, if it persists, changes in the dosage. This process may require more visits to the hospital during the first weeks to determine the appropriate dose, which should be taken into account in the elderly population.

In addition to haematological toxicities, patients who received palbociclib developed more infections, although they were grade 1–2, in addition to reporting more fatigue (Table 4). Regarding quality of life, the surveys evaluated in PALOMA-2/3 showed similar results for the entire population, regardless of age.

#### Ribociclib

Ribociclib is another selective CDK4/6 inhibitor that, when used in combination with hormonal therapy, has shown a significant increase in PFS and OS compared to hormonal monotherapy alone in patients with HR-positive, HER2-negative MBC.

MONALEESA-2 is a phase III trial comparing the combination of ribociclib and letrozole with letrozole monotherapy as the first line of treatment [33, 43]. A total of 668 patients were included, of whom 295 were ≥ 65 years. Regarding the primary endpoint, a higher PFS was obtained in the ribociclib and letrozole arm for both the ≥ 65 years group (HR 0.61; 95% CI 0.39–0.94) and the < 65 years group (HR 0.52; 95% CI 0.38–0.72). An increase in OS was also observed regardless of age (Table 3). The most frequent toxicities observed in the ribociclib arm were haematological changes (neutropenia, leukopenia and anaemia), nausea, diarrhoea, vomiting and fatigue. Regarding the differences in toxicities as a function of age, a higher incidence of anaemia and digestive symptoms was observed in the population ≥ 65 years than in the younger population (Table 4) [32].

MONALEESA-3 is another phase III trial that included the postmenopausal population [36] and compared the combination of ribociclib and fulvestrant with fulvestrant monotherapy in patients who experienced recurrence within 0–12 months after receiving neo/adjuvant treatment and in metastatic patients who had received ≤ 1 line of endocrine treatment for MBC. A total of 726 patients were included; the median age was 63 years (range 31–89), and 47% (*N* = 339) were ≥ 65 years. In this study, an increase in PFS was also observed in the ribociclib arm, with consistent data in all age groups. The HR of patients ≥ 65 years was 0.59 (95% CI 0.43–0.81); in patients < 65 years, it was 0.60 (95% CI 0.45–0.81).

In a subsequent joint analysis of the MONALEESA trials, it was observed that adverse events occurred primarily in the first 3 months of treatment and could be managed well with changes in the administered doses. The efficacy of the treatment was maintained regardless of the intensity of the administered dose [44]. The association of ribociclib use with an increased risk of QT interval prolongation should be considered when selecting this drug. Given the frequent polypharmacy in elderly patients, it is important to review each patient’s usual medications before starting ribociclib.

#### Abemaciclib

Abemaciclib is a potent oral selective CDK4/6 inhibitor. The phase II clinical trial MONARCH-1 [45], which had a single arm, and the phase III clinical trials MONARCH-2 and MONARCH-3 [34, 37] have shown that abemaciclib in combination with AI or fulvestrant increases PFS and OS in patients with HR-positive, HER2-negative MBC. However, available evidence for elderly patients is still limited.

The MONARCH-2 trial included 669 patients [37], 37% (*N* = 245) of whom were > 65 years. The MONARCH-3 study included 493 patients with a median age of 63 years (range 32–88) [34]. Neither of the two trials identified significant differences in PFS between patients > 65 years compared to younger patients (≥ 75 years: HR 0.62; 95% CI 0.34–1.11; 65–74 years: HR 0.63; 95% CI 0.43–0.94; < 65 years: HR 0.52; 95% CI 0.40–0.68; interaction *p* = 0.695 in MONARCH-2, and ≥ 75 years: HR 0.54; 95% CI 0.26–1.13; 65–74 years: HR 0.64; 95% CI 0.40–1.02; < 65 years: HR 0.48; CI 95% 0.35–0.67; interaction *p* = 0.634 in MONARCH-3).

Joint efficacy and toxicity data from the MONARCH-2 and MONARCH-3 trials according to age groups were recently published [46]. The safety data of 1152 patients were evaluated; these data included 156 patients (35%) from MONARCH-2 and 148 (45%) from MONARCH-3 aged ≥ 65 years, of whom 41 (9%) from MONARCH-2 and 42 (13%) from MONARCH-3 were ≥ 75 years of age. The combination of abemaciclib and endocrine therapy demonstrated a tolerable safety profile and a benefit in terms of consistent efficacy for all age subgroups, which supports the use of this combination in older patients. No new safety findings were identified in older patients treated with abemaciclib; therefore, it is not necessary to adjust doses based on patient age alone. The main adverse effect associated with abemaciclib is diarrhoea, which can reach grade 3–4 in 19% of the cases, without differences among age subgroups. This adverse effect is especially important in the elderly population due to the associated risk of dehydration if it is not adequately treated. Grade 3–4 neutropenia with abemaciclib use is observed in approximately 25% of the cases; this incidence is lower than that reported for other cyclin inhibitors (Table 4).

#### Observational studies in clinical practice

Observational studies in routine clinical practice (real-world data [RWD]) provide efficacy and safety data for population groups that are usually excluded from clinical trials, such as older patients or patients with comorbidities. RWD studies of CDK4/6 inhibitors, especially palbociclib and ribociclib, have been conducted. One of the most extensive of these studies is the American Flatiron study [47, 48], which compared letrozole and palbociclib with letrozole monotherapy in routine clinical practice. It included more than 1400 women, with a median age of 66 years (range 58–79), of whom 20% of those who received palbociclib were ≥ 75 years old. In this study, PFS and OS did not differ between patients > 75 years and younger patients. In another American series published by Kish et al*.*, 763 patients with a median age of 64 years were evaluated; among these patients, 50% were < 65 years old, and 21% ≥ 75 years old. In this study, the efficacy and toxicity results, as well as the dose reductions, were similar to those obtained in the PALOMA-2 and PALOMA-3 trials.

In the European context, the most important series and the one that provides the most data in the elderly population is that of the ΗeLLENIC Cooperative Oncology Group (HeCOG) [49], which evaluated 365 patients who had received palbociclib or ribociclib in combination with hormonal therapy. The median age was 61 years (range 34–93), and 12% of the patients were ≥ 75 years of age. The toxicity observed in these patients was similar to that of the younger patients (8 patients, 19%, experienced a grade 3–4 adverse event), and dose reductions or interruptions were not higher in patients ≥ 75 years. The PFS of patients ≥ 75 years was 10.9 months (95% CI 3.1–24.2) when they received the combination of a CDK4/6 inhibitor and hormonal therapy as the first line of treatment and 7.5 months (95% CI 4.5–NR) when they received it as the second line or a subsequent line (*N* = 23). The median OS was 24.2 months (95% CI 10.9–24.2) among those who received this combination as the first line of treatment and has not yet been determined in patients who received this combination as the second line or as a subsequent line.

Therefore, at present, the data obtained from RWD studies confirm that the efficacy and toxicity of CDK4/6 inhibitors are similar to those observed in randomized clinical trials and that the results observed in elderly patients are maintained.

Recently, preliminary data from the French prospective study PALOMAGE were reported [50]. This is a real-life study in patients older than 70 years who received hormonal therapy and palbociclib as treatment for advanced hormone-sensitive or hormone-resistant breast cancer. A total of 407 patients with a median age of 79 years were included, and 15% were older than 85 years. A total of 76% of the patients began treatment with full doses of palbociclib (125 mg), and 63% of these patients were older than 80 years. The most frequent toxicities were neutropenia (43%), anaemia (18%), asthenia (16%) and thrombocytopenia (14%). The incidence of grade 3–4 adverse events related to palbociclib was 40% in patients < 80 years and 31% in patients ≥ 80 years. Dose reduction occurred in 23% of the patients. A total of 42% of the patients discontinued treatment temporarily or permanently. The dose reduction that occurred in 30% of the patients older than 80 years could explain the lower incidence of grade 3–4 adverse events.

### Evidence regarding mTOR inhibitors

Everolimus is a mTOR inhibitor. The PI3K-AKT-mTOR pathway regulates cell growth, proliferation and survival. Its activation has been related to resistance to endocrine treatment. The BOLERO-2 study included patients with disease that was refractory to letrozole or anastrozole who had recurrence in the first 12 months after completing the adjuvant treatment or who progressed to advanced disease in the first month after completing treatment [51]. More than 700 women (38% ≥ 65 years and 23% ≥ 70) with bone or visceral disease were randomly allocated to receive a combination of everolimus and exemestane or exemestane monotherapy. The median PFS was better in the group that received the combination therapy (7.8 vs. 3.2 months; HR 0.45; 95% CI: 0.38–0.54; *p* < 0.0001) [52].

This is one of the few studies to break down the baseline characteristics of patients according to age. Older patients had a higher proportion of visceral than bone involvement [53]. Age (< 70 vs. ≥ 70 years) affected both the intensity of the doses received (8.9 vs. 7.2 mg/day) and the mean duration of exposure to everolimus (33.8 vs. 23.2 weeks) and exemestane (36.1 vs. 27.4 weeks) when the two treatments were administered in combination. Toxicity was similar in both arms (pruritus and diarrhoea), and there was a lower incidence of stomatitis (49% vs. 62%) in patients ≥ 70 years than in patients < 70 years. The rates of discontinuation or dose reduction of everolimus were similar (67% in patients ≥ 70 years and 67% in patients < 70 years), but a higher percentage of discontinuations related to adverse events was observed in patients ≥ 70 years (17%) than in those < 70 years (6%) [54]. The most frequent adverse events in this subgroup were weight loss, dyspnoea, anorexia, asthenia, impaired renal function and urinary infection. However, the relative risk reduction for PFS was similar (56% and 55%) for patients < 70 and ≥ 70 years, respectively [54]. When everolimus was used as a second-line treatment, the median PFS in elderly patients (≥ 70 years) was 1.5 months for monotherapy and 6.8 months for combination therapy, compared to 4.0 and 8.1 months, respectively, in patients < 70 years. The benefits of combined therapy were observed regardless of patient age, although they may not be representative of the real population since the included patients had an ECOG functional status of 0–1. Patients with an ECOG status of 2 represented 1.5% of those < 70 years and 9.3% of those ≥ 70 years; therefore, the safety and efficacy of this treatment in older patients with poor functional status or severe comorbidity are unknown [55]. Therefore, if this treatment is chosen, it is of vital importance not only to provide advice on the management of stomatitis or respiratory symptoms but also to perform an exhaustive prior review of the patient's comorbidities.

### Evidence regarding PI3K inhibitors

Alpelisib is a specific inhibitor of phosphatidylinositol-4,5-bisphosphate 3-kinase catalytic subunit alpha (PI3KCA) and has demonstrated antitumour activity in preclinical models. The phase III SOLAR-1 study compared the combination of alpelisib and fulvestrant with combined placebo and fulvestrant in patients with HR-positive, HER2-negative MBC who progressed during or after treatment with an AI [56, 57]. A total of 572 patients were included, with a median age of 63 years (range 25–92). In the cohort of patients with *PI3KCA* mutations, the median PFS was 11.0 months in the alpelisib and fulvestrant arm (95% CI 7.5–14.5) versus 5.7 months (95% CI 3.7–7.4) in the placebo and fulvestrant arm (HR 0.65; 95% CI 0.50–0.85; *p* < 0.001). Of the 284 patients who received alpelisib, 117 (41%) were ≥ 65 years old, and 34 (12%) were ≥ 75 years old. No differences were found in terms of efficacy in patients aged ≥ 65 years compared to younger patients. The most relevant toxicities observed with alpelisib were hyperglycaemia and diarrhoea. There was a higher incidence of grade 3–4 hyperglycaemia in patients ≥ 65 years (44%) than in patients < 65 years (32%), although it was not necessary to adjust the dose in patients > 65 years [58].

### Evidence regarding PARP inhibitors

The orally administered poly(ADP ribose) polymerase (PARP) inhibitors olaparib and talazoparib have been shown to be effective in patients with HER2-negative MBC and germline mutations in *BRCA1/2* in corresponding phase III studies.

The OlympiAD study demonstrated an increase in PFS in favour of olaparib compared to chemotherapy (capecitabine, eribulin or vinorelbine) (7.0 vs. 4.2 months, respectively; HR 0.58; 95% CI 0.43–0.80; *p* < 0.001). However, more recent data indicate that while the administration of olaparib did not significantly influence OS (19.3 vs. 17.1 months; HR 0.90; 95% CI 0.66–1.23; *p* = 0.513), it positively affected patients’ quality of life [59].

The EMBRACA study compared the administration of talazoparib with the chemotherapy regimen chosen by the investigator [60]. Patients who had been previously treated with platinum were included. PFS was better with the use of talazoparib (8.6 vs. 5.6 months; HR 0.54; 95% CI 0.41–0.71; *p* < 0.0001). However, when the data were updated, no benefit in OS was observed [61]. The population aged ≥ 65 years in these studies comprised less than 10% of the sample, and therefore, the conclusions for this population group are limited.

### Evidence regarding hormonal monotherapy

Currently, the combination of a CDK inhibitor with endocrine therapy is the standard first-line treatment for patients with luminal MBC. However, for frail patients who are polymedicated or have little social or family support and who are not candidates for this treatment, hormonal therapy administered as monotherapy may be a reasonable option [62]. An AI is the most commonly used first-line treatment in these cases. The superiority of AIs over tamoxifen in the postmenopausal population has been demonstrated in various clinical trials [63, 64], although in patients ≥ 70 years of age, it has only been demonstrated in a single clinical trial with letrozole [65]. Patients who have benefited from first-line hormonal treatment and had good tolerance but developed disease progression after a lasting response can benefit from second-line hormonal treatment with other hormonal therapies, such as tamoxifen, fulvestrant and megestrol acetate or exemestane if a nonsteroidal AI has been administered as the first line of treatment [66, 67]. The optimal endocrine therapy sequence is unclear and will depend on which agents have been previously used, the duration of the response obtained, the disease burden and patient preferences [26, 68].

### Evidence regarding chemotherapy

The justification for administering chemotherapy as the first-line treatment for elderly patients with luminal MBC is the existence of a visceral crisis; the justification for its use in successive lines of treatment is refractoriness to hormonal treatment [26, 69]. Two decades ago, Christman et al*.* demonstrated that patients aged > 70 years who underwent chemotherapy treatment for metastatic disease obtained a benefit similar to that of younger patients in terms of response rates and PFS [70]. The difficulty for these patients lies in the fact that chronological age or functional status alone does not determine the risk of developing toxicity, and, therefore, it is essential to find a reliable method for predicting this risk [20, 21]. In this context, some recommendations must be taken into account before administering chemotherapy to elderly patients: (i) single-agent chemotherapy is preferred over combination regimens, which are usually more toxic; (ii) the use of oral metronomic regimens is recommended if it is not necessary to obtain a rapid response; (iii) weekly regimens should be prioritized; and (iv) it is advisable to administer cytotoxic agents that have a favourable safety profile, such as taxanes, liposomal doxorubicin, gemcitabine, capecitabine or vinorelbine [71].

Most published studies regarding this condition are small phase II clinical trials and retrospective studies in which chemotherapy regimens were administered as monotherapy [72–75]. Given that chemotherapy in this situation has a palliative objective, it is essential to maintain patient quality of life by keeping toxicity to a minimum.

### Breast cancer in elderly men

Breast cancer in men is more common in the elderly population than in younger populations, and in most cases, high HR expression and HER2 negativity is observed [76]. With respect to the therapeutic management of advanced disease, the same approach that is used for women should be used. Hormonal treatment is administered as monotherapy or in combination with CDK4/6 or mTOR inhibitors. Tamoxifen is usually administered, but if an AI is prescribed, it must be accompanied by a gonadotropin-releasing hormone (GnRH) agonist analogue. If chemotherapy is selected, the same drugs and treatment regimens that are used for the female population should be offered [77].

### General considerations in the elderly population

Ageing involves pharmacokinetic and pharmacodynamic alterations that modify drug bioavailability, increasing the possibility of adverse effects [78]. Older adults with cancer should be offered the best available treatment option based on efficacy and tolerability, and in selecting treatments, the following factors should be considered:

(i) Concomitant treatments that the patient is receiving should be considered to avoid drug interactions. Polypharmacy is one of the most common problems in the elderly population, and therefore, it is important to review interactions between drugs that are being taken and drugs that may be prescribed, including interactions with foods that can increase toxicity or decrease efficacy [79]. For example, the SOLTI group has developed a tool called Cyclib-TOOL (www.cyclibtool.org) that reports the possible interactions between CDK4/6 inhibitors and other commonly used drugs and offers safe therapeutic alternatives.
(ii) Comorbidities such as liver and kidney failure and/or heart rhythm disorders should be considered. Doing so will require an exhaustive review of the metabolism and excretion of the drug to be prescribed, as well as close monitoring. Similarly, in diabetic patients, special care should be taken to avoid decompensation when corticosteroids are prescribed as antiemetics or if the drug causes diarrhoea and the patient is not adequately hydrated.
(iii) In the case of oral treatments, adherence should be closely monitored, since it can be more easily compromised in older patients, specifically in the presence of relevant risk factors such as cognitive decline, multimorbidity, polypharmacy and little social or family support. These treatments will only be effective if adherence is optimized. Barriers to good adherence must be identified and proactively managed in a multidisciplinary environment [80].
(iv) Last, it is important to maintain patient quality of life and functionality [81, 82]. Treatment for MBC is palliative; therefore, symptom control and quality of life play a fundamental role. In the case of the elderly population, physicians should look beyond the tumour stage and pay greater attention to the patient, evaluating his or her functional status, comorbidity, polypharmacy, mobility, nutritional status, mental health, cognitive status, social situation and quality of life. For example, asthenia is one of the adverse events with the greatest impact on older people, but others are also relevant, including neuropathy (which increases the risk of falls), nausea and frequent trips to the hospital alone or with a relative [83].

## Recommendations established in the major clinical guidelines

In 2018, the SEOM published general recommendations for the management of elderly patients with cancer [11]. This guideline highlights the importance of the correct performance of a CGA using the pathways and resources available at each institution to produce an individualized treatment proposal, and it notes that multidisciplinary intervention is a cornerstone of this process. Some general recommendations should be considered in the oncological treatment of any elderly person, such as providing intervention specifically for the major geriatric syndromes detected in the CGA and selecting the least toxic chemotherapy regimens for the patient. In turn, special attention should be given to the early prevention of adverse events and to adequately controlling symptoms and monitoring social support, which is essential for proper treatment planning. Similarly, elderly patients with cancer require follow-up that is adapted to their comorbidities, which can influence the behaviour of the disease, the response to treatment and the type of treatment administered in various ways.

SIOG, together with the European Society of Breast Cancer Specialists (EUSOMA), also published recommendations for treating BC in elderly patients [13]. Given that these recommendations were published in 2012, there are no specific indications for the use of CDK4/6 inhibitors, but some generalities similar to those described above are outlined. These guidelines suggest prioritizing hormonal treatment in patients with luminal MBC and consider this the option of choice for elderly patients, with chemotherapy reserved for cases in which the disease is hormone resistant or rapidly progressive. Dose reductions and regimens changes are controversial but should be considered according to each drug and its toxicity [13].

Recently, the 5th International Consensus of the European School of Oncology (ESO)-ESMO on the treatment of MBC was published. There is no special mention of elderly patients in this guideline, but it established that age should not be the only reason for not offering an effective treatment to elderly patients, nor should it determine the intensity of treatment. The combination of a CDK4/6 inhibitor with hormonal therapy in patients with luminal MSC is highlighted as the first option due to its observed efficacy, but no age group is specified for this treatment [26].

In the latest update of the guidelines of the National Comprehensive Cancer Network (NCCN) for elderly patients with cancer, the importance of performing an accurate CGA that guides decision-making for patient when a concern regarding tolerance to treatment exists is emphasized. This process allows the elderly patient’s problems and needs in different areas of life to be detected and quantified. Once these deficits are detected, an intervention strategy should be developed, usually with a multidisciplinary team [19].

The scarcity of robust data on the treatment of BC in elderly patients prevents these recommendations from reaching an evidence level of evidence 1 (Table 5), but experts agree that age alone should not influence any aspect of treatment and that all decisions must consider other aspects, such as biological age, life expectancy, the risks and benefits derived from each treatment and patient preferences.

**Table 5:** Recommendations by the major clinical guidelines for the treatment of elderly patients with luminal MBC

Clinical guideline	General recommendations
SEOM, 2018 [11]	Perform a CGA—importance emphasized Conduct a multidisciplinary intervention Be aware of concurrent major geriatric syndromes Avoid concomitant chemotherapy and radiotherapy regimens Consider the administration of metronomic chemotherapy Control symptoms and toxicity as early as possible
SIOG-EUSOMA, 2012 [13]	Be aware that no specific indications for the use of CDK4/6 inhibitors have been made since the guideline was published in 2012 Consider hormonal treatment as a priority Consider other general recommendations similar to those described in the SEOM section
ESO-ESMO, 2020 [26]	Consider the combination of a CDK4/6 inhibitor with hormonal therapy as the first option Note that treatment is not specified according to age group
NCCN, 2021 [19]	Perform a CGA—importance emphasized when concerns about treatment tolerance exist Determine patient deficits in different areas to develop an appropriate multidisciplinary intervention strategy

*MBC* metastatic breast cancer, *ESMO* European Society of Medical Oncology, *ESO* European School of Oncology, *EUSOMA* European Society of Breast Cancer Specialists, *NCCN* National Comprehensive Cancer Network, *SEOM* Spanish Society of Medical Oncology, *SIOG* International Society of Geriatric Oncology, *CGA* comprehensive geriatric assessment, *CDK4/6* cyclin-dependent kinase 4 and 6

## Conclusions

The majority of patients diagnosed with luminal MBC who are seen in oncology consultations are elderly. In general, the efficacy of cancer drugs in elderly patients is similar to that described in younger populations, although the toxicity may be somewhat higher. Treatment selection for the elderly population should consider the patient’s baseline status as well as the expected benefit and toxicity of each treatment. The impact of treatment toxicity on the patient’s quality of life and functionality should also be taken into account.

CDK4/6 inhibitors such as palbociclib, ribociclib and abemaciclib are safe and effective drugs for the elderly population. Any of the three drugs in combination with hormonal treatment have been shown to prolong PFS in elderly patients in a manner similar to the benefit obtained by the younger population when compared with the administration of hormonal therapy as monotherapy. The toxicity of the combination of a CDK4/6 inhibitor with hormonal therapy in the elderly population is somewhat higher than that in the younger population, but it is manageable. Therefore, it is not recommended to reduce doses at the beginning of treatment simply because of the age of the patient. The selection of a CDK4/6 inhibitor will depend on the toxicity profile of the drug, the patient’s comorbidities and possible interactions with other drugs that the patient is taking.

Regarding mTOR (everolimus) and PI3K (alpelisib) inhibitors, although the evidence on their use in older patients is very limited, their efficacy in these patients seems to be similar to that in younger patients, but the toxicity is greater. Chemotherapy, when indicated, is best administered as monotherapy and in weekly regimens, always taking into account the toxicity profile of each drug.

It is important to note that the main national and international clinical guidelines agree that the chronological age of the patient alone should not guide therapeutic decisions and recommend performing a CGA whenever possible before establishing treatment.

## Data Availability

Not applicable.
